# Cognitive and Plasma Biomarker Profiles of Older Adults with Probable Alzheimer's Disease in Kinshasa, Democratic Republic of Congo

**DOI:** 10.1002/alz70856_103374

**Published:** 2025-12-25

**Authors:** Emmanuel Epenge, Jean N Ikanga

**Affiliations:** ^1^ Global Brain Health Institute, University of California San Francisco, San Francisco, CA, USA; ^2^ Protestant, KINSHASA, KINSHASA, Congo (The Democratic Republic of the); ^3^ Emory University, Atlanta, GA, USA; ^4^ University of Kinshasa, Kinshasa, Kinshasa, Congo (The Democratic Republic of the)

## Abstract

**Background:**

Many studies in Western countries have established cognitive and plasma biomarker profiles of Alzheimer's disease (AD) and related dementias for diagnostic accuracy, monitoring disease progression, personalized medicine, and research advancements. Due to the lack of such profiles in low‐ and middle‐income countries, our study presents the cognitive and biomarker profiles of older Congolese adults with probable AD.

**Method:**

This cross‐sectional, descriptive, and analytic study involved 114 participants aged 50 years and older, including 56 older adults with probable Alzheimer's disease (pAD) and 58 healthy controls (HC) recruited from community environments (churches, associations of older adults) between October 2019 and December 2022. The DSM‐5‐TR, the CSID, the AQ, and the African Neuropsychology Battery (ANB) were used to differentiate between the two groups (pAD vs. HC). Analysis of variance (ANOVA) was used as the statistical test, with *p* < 0.05 as the threshold for statistical significance.

**Result:**

The average age was 73 ± 8 years with a sex ratio of 1M:1F. Cognitive deficits were more significant in patients with Alzheimer's disease than in HC, particularly in verbal memory, visuospatial memory, language, facial perception, and executive functions (problem‐solving) (*p* < 0.05). A reduced Abeta 42/40 ratio, increased amyloid probability score (APS), and Apoe4 were predominant in pAD patients compared to HC (*p* < 0.05). Abeta 40, Abeta 42, and ptau 181 did not show significant differences between the two groups.

**Conclusion:**

These findings are similar to previous Western studies, which have highlighted the same cognitive and biomarker profiles

